# Identification and Quantification of Phenolic Compounds from Mexican Oregano (*Lippia graveolens* HBK) Hydroethanolic Extracts and Evaluation of Its Antioxidant Capacity

**DOI:** 10.3390/molecules26030702

**Published:** 2021-01-29

**Authors:** María del Carmen Cortés-Chitala, Héctor Flores-Martínez, Ignacio Orozco-Ávila, Carolina León-Campos, Ángela Suárez-Jacobo, Mirna Estarrón-Espinosa, Irma López-Muraira

**Affiliations:** 1 TecNM/ITTlajomulco. Km. 10 Carretera Tlajomulco-San Miguel Cuyutlán, Tlajomulco de Zúñiga 45640, Jalisco, Mexico; mcarmencc29@gmail.com (M.d.C.C.-C.); caroleon03@hotmail.com (C.L.-C.); lopezmuraira@hotmail.com (I.L.-M.); 2 Tecnología Alimentaria, Centro de Investigación y Asistencia en Tecnología y Diseño del Estado de Jalisco, A.C. Camino Arenero 1227, el Bajío del Arenal, Zapopan 45019, Jalisco, Mexico; iorozco@ciatej.mx (I.O.-Á.); mestarron@ciatej.mx (M.E.-E.); 3 Biotecnología Industrial. Centro de Investigación y Asistencia en Tecnología y Diseño del Estado de Jalisco, A.C. Camino Arenero 1227, el Bajío del Arenal, 45019 Zapopan, Jalisco, Mexico; asuarez@ciatej.mx

**Keywords:** Mexican oregano, *Lippia graveolens*, flavonoids, identification, quantification, antioxidant activity

## Abstract

Plants have been used for thousands of years for various purposes because they have a wide variety of activities with biological significance. Mexican oregano is an aromatic plant of great importance to Mexico and north of Jalisco state as a spice with important economic value. Chromatographic identification and quantification of phenolic compounds and evaluation of their antioxidant activity were important tools to obtain a better characterization of this spice. Phytochemical analysis indicated the presence of flavonoids, triterpenes, saponins, quinones and tannins, the latter at high concentrations. Through chromatographic assays of Mexican oregano extracts, 62 compounds were identified, the major ones being quantified as: taxifolin, apigenin 7-*O*-glucoside, phlorizin, eriodictyol, quercetin, naringenin, hispidulin, pinocembrin, galangin and genkwanin (compound for the first time reported for this species). The results can be useful as a precedent to establish the bases of new quality characterization parameters and they have also suggested that Mexican oregano contains a wide variety of compounds with untapped importance for the development of new high value-added products.

## 1. Introduction

Aromatic plants and spices have been used since ancient times for several purposes. Their biological potential is related to compounds known as plant secondary metabolites or phytochemicals [1,2]. These compounds are produced by plants in response to environmental stimuli such as defense and competition between plants systems and as attractants of beneficial organisms. These compounds have been grouped as phenolics, alkaloids and terpenes, among others [3,4]. Similarly, the therapeutic and pharmacological properties of plants as well as the biological antioxidant potential are attributed to these molecules [1,2,3,4,5].

The composition and concentration of secondary metabolites present in plants depend on their genotype, climatic factors, altitude, harvest time and their state of growth [6]. The health promoting potential of phytochemicals is of great interest for the pharmaceutical and food industries due to their biotechnological applications [5,7]. The presence and characterization of phytochemicals in aromatic plants and spices stimulate the use and commercialization of value-added products [1,3,5,7].

The growing interest of the food industry for phytochemicals has been increasing due to the disapproval and disuse of chemical additives in food and their processes [8,9,10,11]. As an example, the natural antioxidants from *Rosmarinus*, carnosol, carnosic acid and rosmarinic acid, have been more effective than butylated hydroxytoluene (BHT) and butylated hydroxyanisole (BHA) to protect fats and products with a high-fat content from oxidation [12].

The dry leaves of Mexican oregano (*Lippia graveolens* H.B.K.) are used mainly as a condiment of numerous traditional dishes in the Mexican cuisine. It is known that these leaves have the capacity to naturally conserve and enhance the flavor of foods [8,9,10,11]. Industrially, the main product of Mexican oregano is the essential oil, which has been widely studied and characterized [10,13]. Internationally, this aromatic product has been used as raw material for the pharmaceutical, food and cosmetic industries [14,15,16,17,18]. However, their important content of antioxidant non-volatile compounds highlight Mexican oregano as a potential food additive [8,9,10]. Regionally, producers usually sell the collected leaves without giving any added value [15]. Few studies have focused on the characterization of the non-volatile compounds of Mexican oregano and even fewer have focused on the possible changes in the composition given by the collection area [6]. The present work aims to characterize and quantify the main phenolic compounds present in hydroethanolic extracts of Mexican oregano from three different localities and to evaluate their antioxidant capacity.

## 2. Results and Discussion

### 2.1. Physico-Chemical Analysis

The botanical analyses of leaves, stems, and flowers identified the plant samples collected as *Lippia graveolens* H.B.K. However, the density and size of the villi and glands present in their flowers suggest a different state of maturity. The plant from Mezquitic (OM) is the younger sample as it shows the lower density of villi and small glands. According to this, the oregano from Huejuquilla (OH) is referred as the most mature, due to its large number of villi and glands and larger size of these. Oregano from Colotlan (OC) had the highest yield of essential oil (EO), followed by oregano form Mezquitic and finally oregano from Huejuquilla (Table 1).

Differences between EO content may be due to the region of harvest as well as the age of the plant, as reported by Pereira for *Lippia gracilis* [6].

### 2.2. Phytochemical Analysis

Phytochemical analysis shows the presence of several chemical families of compounds in the plant (Table 1). As can be observed, there are important differences in qualitative composition among the analyzed samples. The observations were recorded based on the intensity of the color change as strongly present, moderately present, weakly present or not detected. Flavonoids were detected in the three samples, the coloration was higher in the oregano from Huejuquilla, followed by the oregano from Colotlan and in lower concentration in the Mezquitic oregano. Terpenes and quinones were observed only in plants from Mezquitic and Colotlan. Oregano from Colotlan showed a higher concentration of steroids, followed by OM and OH, respectively. The saponin test was positive only for OH and OC. Tannins were detected in all samples in high concentrations while the presence of coumarins was negative for all of them.

The differences found in the qualitative composition between the samples collected from different regions could be explained by several factors. In plants during the biosynthesis of secondary metabolites, a compound structure can be the precursor of a variety of others. Thus, the differences in the qualitative composition will depend on climatic conditions, phenological status and ecological stress in which the plant was developed [6]. These findings are important because the functional properties of spices in traditional and herbal medicine have been related to the presence of certain compounds families. The presence of flavonoids, a well-known family of polyphenols, because of their antioxidant properties may be related to the antitumor activity attributed to Mexican oregano [2]. Tannins are astringent in nature and useful in the treatment of intestinal disorders such as diarrhea and dysentery, properties also attributed to this *Lippia* species [8,19]. Saponins are surfactants that produce hemolysis of red blood cells and in recent years, they have become important because they are used as raw material for the synthesis of steroidal hormones used in medicine, hence oregano is used as an estrogenic and abortive agent [2]. Mexican oregano has been used in the treatment of respiratory allergies [8,19], this may be due to the ability of terpenoids to improve lung function in respiratory treatments [20]. On the other hand, anthraquinones are characteristic of *Verbenaceae* family and they function as purgative and coloring agents [21].

### 2.3. Total Phenolics and Antioxidant Capacity

The analysis of Folin–Ciocalteu (Table 2) showed that the content of polyphenolic compounds in the Mexican oregano samples varied from 4.28 to 4.54 mg GAE/mL Ex. Likewise, the phenolic concentrations (expressed on a dry basis): OH = 96.72, OM = 95.74, OC = 99.71 mg GA/g DW, are higher than reported in herbs like basil (19.5), chili (9.2), coriander (17.3), garlic (2.3), ginger (13.7), lemongrass (13.6), parsley (7.0) and Spanish oregano (88.5) [22]. The antioxidant capacity of the Mexican oregano extracts showed values from 7.54 to 8.80 and from 1.65 to 1.67 mg Tx Eq/mL Ex for DPPH and ORAC, respectively. The antioxidant activity (DPPH) found in Mexican oregano samples are higher than those described for Spain oregano (1.14 mg/mL) [22]. The antioxidant potential and phenolics content of the three plant materials of *Lippia graveolens* did not show significant differences among them.

The quantification of total phenols includes flavonoids, tannins and quinones and the small differences found may be due to the concentrations and dissimilar proportions of these compounds between them. The differences between the ORAC and DPPH values may be due to the reaction mechanism of each test. While the determination by DPPH is based on the transfer of electrons, the basis of the ORAC reaction is the transfer of protons. Similarly, the DPPH test quantifies the antioxidant capacity measured at a given time, while the ORAC test measures the area under the curve (AUC), which combines the time of inhibition and the degree of inhibition of free radicals by an antioxidant or an extract a certain concentration. These results can be used to establish the basement for the standardization and characterization of this resource for future formulations of food antioxidant additives.

### 2.4. Identification and Quantification of Polyphenols by LC-ESI-QTOF-MS

**Figure 1:**
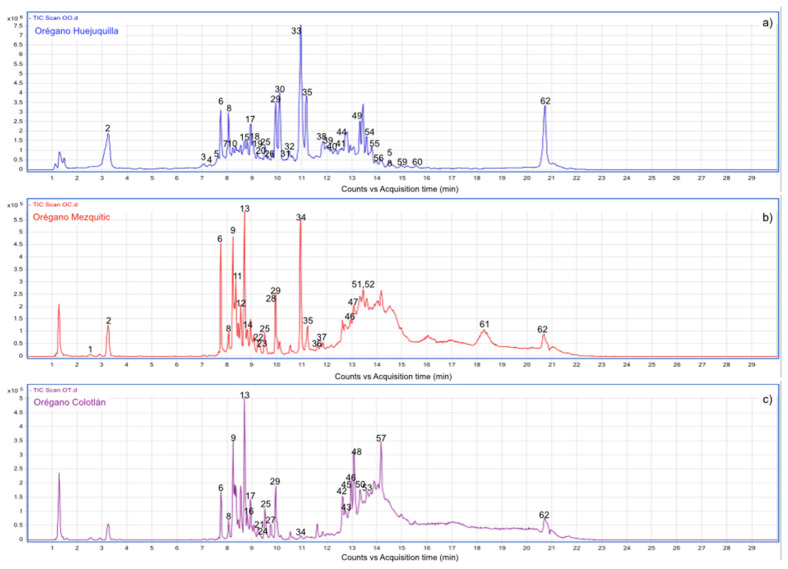
Representative chromatogram of phenolic compounds of Mexican oregano extracts from different regions of Jalisco state. (**a**) Oregano from Huejuquilla, (**b**) oregano from Mezquitic, (**c**) oregano from Colotlan.

The data acquired from LC-ESI-QTOF-MS allowed the identification a total of 62 different compounds. Of which, 41 are present in OH, 33 in OM and 24 in OC, presenting some common compounds in two or all samples analyzed. Of the total, *6*, *8*, *9*, *10*, *11*, *12*, *15*, *16*, *17*, *20*, *21*, *25*, *28*, *29*, *30*, *32*, *33*, *34*, *35* and *62* peaks are common in all three samples, *1* and *2* were found in both the OH, and OM samples and *13*, *24*, *45*, *46* and *52* are common in the Mezquitic and Colotlan samples. On the other hand, peaks *23*, *10* and *7* were identified only for OH, OM, and OC, respectively. Figure 1 shows the chromatograms of the total signal obtained from the crude extracts of the different samples. The molecular ions and retention times of each peak are listed in Table 3.

With the retention times and pseudomolecular ions of the standard substances, the peaks *15*, *16*, *17*, *20*, *25*, *29*, *30*, *33*, *34* and *35* were identified as taxifolin, cosmoside, phlorizin, eriodictyol, quercetin, naringenin, hispidulin, pinocembrin, galangin and genkwanin, respectively.

With the exception of genkwanin, all the compounds identified by standards compounds were reported in the works carried out by Lin et al. [9] with methanolic extracts of Mexican oregano. Genkwanin has previous reports in *Lippia rigida* [23] and *Rosmarinus officinalis* [12], however, to our best knowledge, this represents his first report for *Lippia graveolens* HBK. Furthermore, this is the second report of phlorizin in *Lippia graveolens* HBK and in the *Verbenaceae* family [9]. Taxifolin was also identified in *L. salviaefolia*, *L. balansae*, *L. velutina* and *L. sidoides* [24]. Apigenin 7-*O*-glucoside (cosmoside) has been reported in *L. balansae*, *L. velutina* and *L. sidoides* [24] and in some commonly used spices such as *Rosmarinus officinalis* [12,25], *Thymus vulgaris* and *Origanum vulgare* [25]. Eriodictyol and hispidulin were also identified in *Origanum vulgare* [26] and *Rosmarinus officinalis* [12], respectively. The last one has been identified in *Lippia* genus, specifically in *Lippia alba* carvoneifera, *Lippia alba* citraleifera, *Lippia sidoides* Mart y *Lippia alba* myrceneifera [27] Quercetin has previous reports in *Lippia salviaefolia*, *L. balansae*, *L. velutina* and *L. beenises* [24] and other spices: rosemary (*Rosmarinus officinalis*), thyme (*Thymus vulgaris*), oregano (*Oreganum vulgare*), cinnamon (*Cinnamomun zeylanicum*), cumin (*Cuminum cyminum*) and laurel (*Laurus nobilis*) [25]. Naringenin has been identified in *Lippia salviaefolia*, *Lippia balansae* [24], *Oreganum vulgare* [26] and *Laurus nobilis* [25]. Table 4 summarizes uses and properties of these compounds in previous reports.

The identification of previously reported pseudomolecular ions allowed identifying 12 compounds. Peaks *9*, *10* and *11* are common in all the samples, while peaks *5*, *7*, *26* and *32* are present only in OM. Peaks *12* and *52* are present in the OH sample. Peak *13* is in both OM and OC, while peaks *28* and *35* are common in OH and OM.

The pseudomolecular ion of peak *10* (463) has been reported as 6-hydroxyluteolin-7-*O*-hexoside, a glycosylated flavone previously reported by Lin et al. [9] in samples of the same species. This compound has also been found in *L. salviaefolia*, *L. balansae*, *L. velutina* and *L. sidoides* [24]. The pseudomolecular ions of the peaks *30* (m/z = 299) and *32* (m/z = 313) are similar to that established by the luteolin standard (m/z = 285.0405, tetrahydroxylated flavone). However, the first ion (m/z = 299) presents 14 amu additional and in the second (m/z = 313), the difference is 28, which is a substituent -CH_3_ in peak *30* and a double methylation in the case of peak *32*. Flavones reported with these pseudomolecular ions and structures are hispidulin and cirsimaritin, respectively. Both have been previously reported in Mexican oregano [9], moreover, hispidulin was also reported in rosemary [12]. Similarly, the pseudomolecular ion of peak *28* (273.0763) differs from the ion established by phlorizin (peak *17*, 435.1297) by 162 amu, which implies the presence of a glucose in the standard. The aglycone of this compound corresponds to a trihydroxylated dihydrochalcone, phloretin. There are previous reports of this compound in *L. graveolens* [7], *L. salviaefolia*, *L. balansae*, *L. velutina* and *L. beenises* [24]. On the other hand, the m/z value of peak *13* only differs 16 amu from the same standard, which concludes the presence of 3-hydroxylphloretin 6′-*O*-hexoside also reported by Lin et al. [9] in Mexican oregano. The peaks *5*, present in OM, *9* and *11*, present in the all three samples, have pseudomolecular ions corresponding to four stereophors, more specifically; (2*R*)-3,4,5,6-tetrahydroxyflavanone 7-*O*-β-glucopyranoside, (2*S*)-3,4,6-tetrahydroxyflavanone 7-*O*-β-glucopyranoside, (2*R*)-3,4,5,8-tetrahydroxy-flavanone 7-*O*-glycolpyranoside and (2*S*)-3,4,5,8-tetrahydroxy-valvanone 7-*O*-glycolpyranoside. These isomers have been previously reported in *Lippia salviaefolia*, *L. balansae*, *L. velutina* and *L. sidoides* species [24]. Some reports in citrus fruits indicated the presence of an enantiomeric and epimeric mixture of other flavanones. Their respective 7-*O*-glycosides and their different ratios of 2*S*/2*R* isomers have been related to the ripeness of the fruits [40,41]. The pseudomolecular ion of peak *7* has been reported for the compounds of apigenin 6, 8-di-*C*-glucoside [26] in samples of *O. vulgare* and kaempferol 3-*O*-rutinoside [25] in samples of rosemary, thyme, oregano, cumin and laurel. The spectrum of peak *12* showed a pseudomolecular ion of 623.1981 corresponding to a glycosylated phenylpropanoid. Spectrum of peak *12* showed a pseudomolecular ion of 623.1981, Soleo et al. [24] reports on *L. salviaefolia*, *L. balansae*, *L. velutina*, *L. sidoides*, *L. lasiocalycin* and *L. lupilina* this same ion for the isomers verbascoside, isoverbascoside and forsitoside A. The ion established by peak *26* is previously reported as C-hexoside sinapic acid in spices such as rosemary and thyme [25]. Peak *52* shows a pseudomolecular ion of 301.2173, which has been reported by Borrás [12] for salviol diterpene in samples of *Rosmarinus officinalis*. The formula C_16_H_22_O_11_ was obtained from the spectrum of peak *6*. This can belong to three different compounds: asperulosidic deacetyl acetic acid, teveside and monotropein, reported in a wide variety of species. However, the teveside has been reported in *Lippia citriodora* and *Lemon verbena*, both belonging to the *Verbenaceae* family.

Another group of compounds was identified by the formula generation tool of the software. A total of 19 compounds were identified. Peaks *3*, *4*, *18*, *38*, *40*, *55* and *56* were found only in OM; *22*, *23* and *47* were in the Huejuquilla sample and *50*, *53* and *57* were in OC. Peaks *6*, *8* and *21* were common in the total of the samples. Peaks *1* and *2* were located in both OM and OH and only peak *45* was in the samples from Huejuquilla and Colotlan. No matches were found between the Mezquitic and Colotlan samples. Peaks *31*, *36*, *42*, *43*, *44*, *46*, *48*, *49*, *51*, *54*, and *58* could not be identified by any of the previous methods, but they could be related to a chemical structure. It was not possible to identify or associate the peaks *14*, *19*, *24*, *27*, *37*, *39*, *41*, *59*, *60*, *61* and *62* to any structure.

Compounds related to peak *8* (C_18_H_28_O_9_) are (1*R*, 2*R*)-5′-hydroxyjasmonic acid 5′-*O*-β-d-glucopyranoside or its isomer, the tuberonic acid glycoside, these compounds have also been found in *Lippia citriodora* [42]. Peaks *6* and *8* were present in all the three analyzed samples. Formulas C_35_H_52_O_6_ and C_35_H_52_O_5_ were obtained from peaks *50* and *56*, respectively. The first one corresponds to the camaric acid found in *Lantana camara* and *Lantana cujabensis*. The second one belongs to two isomers, lantadene A and lantadene B, both found in *Lantana camara* [28]. The spectrum acquired from peak *21* was present in the three samples of oregano, corresponds to 2-*O*-(3,4-dimethoxybenzoyl) orientin (C_30_H_28_O_14_), a compound reported in flowers of *Trollius ledebourii* [28].

Peaks *1* and *2* were presented in both Mezquitic and Huejuquilla samples. Their spectra provided the following formulas C_21_H_22_O_14_, for peak *1*, and C_16_H_24_O_11_, for peak *2*. The first one can correspond to three isomers: methyl 4,6-*O*-di-*O*-galloyl-β-d-glucopyranoside, methyl 6-*O*-digalloyl-β-d-glucopyranoside I or methyl 6-*O*-digalloyl-β-d-glucopyranoside II. These compounds have been reported in *Sanguisorba officinalis*. The second one belongs to tuliposide F or cachinoside IV, found in species of *Tulipa turkestani* and *Campsis grandiflora*, respectively [28]. Spectra of the peaks *45* (OH and OC), *47* (OH) and *57* (OC), correspond to the same formula (C_30_H_38_O_4_), which may correspond to lancilactone B previously found in *Kadsura lancilimba* [28]. Peak *53* was called grandidone D (C_40_H_48_O_8_). This compound has been previously reported in *Plectranthus grandidentatus* [28]. In the samples analyzed it was only presented in those from Colotlan. Peaks *22* and *23* were only presented in OH. The first one was designated as ikarisoside F (C_31_H_36_O_14_) present in different species of *Epidemium*. Formula C_31_H_30_O_15_ was obtained from spectrum of peak 23. This formula corresponded to 6-*O*-p-hydroxybenzoyliridine, previously located in *Belamcanda chinensis* [28]. Peaks *3* and *4* were designated as 5,7,8-trihydroxycoumarin-5-β-glucopyranoside (Polytrichum formosum) and 1-*O*-(4-Hydroxybenzoyl)-β-d-glucose (*Crocus sativus* pollen and *Luffa cylindrica*), respectively [28]. The spectrum obtained from peak *18* corresponds to 4-methoxyphenyl 1-*O*-β-d-[5-*O*-(3,4-dimethoxybenzoyl)]-apio-furanosyl-(1-6)-β-d-glucopyranoside (C_27_H_34_O_14_), it was found in *Tabebuia impetiginosa*. The formula C_18_H_30_O_3_ (peak *38*) can correspond to two molecules, 13(R)-Hydroxy-octadeca-(9*Z*, 11*E*, 15*Z*)-trienoic acid (*Potamogeton lucens*) and the hygrosforone F (*Hygrophorus persoonii*) [28].

From the spectrum of peak *40* the formula C_18_H_32_O_3_ was acquired, this may correspond to (*S*)-cariolic acid and coronaric acid, found in *Hernandia sonora* and *Chysantemun coronarium* [28], respectively. Peak *55* was named lespedezol B3, compound reported in *Lespedeza homoloba* [28]. The spectra described above were only presented in the samples from Mezquitic.

The formula acquired by peaks *36*, *42* and *46* corresponds to more than 14 compounds, however, all have structure of diterpenic quinone. Similarly, peaks *49*, *51* and *54* coincide in flavonoid structure, with more than 10 possible compounds for each. The formula generated for peak *31* can refer to more than 7 compounds, all with diterpenic taxoid structure. The peaks *43* and *48* refer to more than 23 sesquiterpenes and 7 diterpenes, respectively. The formulas of peaks *44* and *58* have chalcone and anthrone structure, respectively. It was not possible to identify or associate the peaks *14*, *19*, *24*, *27*, *37*, *39*, *41*, *59*, *60*, *61* and *62* to any structure.

Out of the 22 compounds fully identified (12 compounds by m/z and 10 by standard compounds compounds); 13 of them were already reported in the work of Lin et al. [9] in methanolic extracts: 6-hydroxyluteolin 7-*O*-hexoside, taxifolin, 3-hydroxyphloretin 6′-*O*-hexoside, apigenin 7-*O*-glucoside, phlorizin, eriodictyol, quercetin, naringenin, hispidulin, cirsimaritin, pinocembrin, galangin and methylgalangin. The previous reports have a prior fractionation of the extract and therefore a pre-purification of the compounds evaluated. However, the extracts used were obtained through optimal conditions reported by our research group in a previous study to *Lippia graveolens* HBK, focused for obtaining extracts with maximum antioxidant activity [43]. These conditions allowed the extraction of a wide variety of compounds as shown in the chromatographic analysis. The chromatographic method allowed the separation of 62 peaks and the identification of 40 compounds, in comparison to the 23 compounds extracted and identified for the same plant [9]. This provides a more complete screening of the compounds presented in Mexican oregano. The improvement in the results obtained could have been due to the use of a high-performance liquid chromatography coupled to electrospray quadrupole-time of flight mass spectrometry, which has proven to be a valuable detection system for characterizing a wide range of phenolic compounds since it provides high mass accuracy and true isotopic pattern in MS spectra [12].

Quantification of the major phenolic compounds in oregano extracts describes the samples in different ways depending on the compound (Table 5). The content of apigenin 7-*O*-glucoside and quercetin (0.008–0.015 and 0.014–0.018 mg/mL Ex, respectively) does not show significant differences among them. On the other hand, the content of both eriodictyol (0.017–0.044 mg/mL Ex) and galangin (0.003–0.436 mg/mL Ex) show that the oregano samples are significantly different. The concentration of taxifolin shows that the OC sample is different from the others. Additionally, naringin and genkwanin group OC and OH are different from OM. Sample from Huejuquilla was significantly different from others due to its content in phlorizin, hispidulin and prinocembrin. The latter in high concentrations (3.231 mg/mL Ex).

Table 5 shows that the naringenin content in the three samples is lower than those reported for the same specie [9] and *L. sidoides* Mart [44]. However, the pinocembrin and galangin concentrations in the OH sample are higher than those found in previous reports [9].

In general, the evaluation of a crude extract, in the present work, allowed the identification of various structures, not only flavonoids, for example diterpenes (salviol), phenylpropanoids (verbascoside and its isomers) and quinones, resulting in a better knowledge of the type of compounds present in the plant. These results lay the foundations to expand the uses of *Lippia graveolens* H.B.K. beyond its use as spice or for the extraction of essential oil.

Despite the antioxidant capacity is not significantly affected by composition, the potential use of one specific sample can be favored by the interest in some of the major compounds.

Considering that this study was carried out with samples collected only in one year, for a complete characterization of Mexican oregano specie is required to study their composition and antioxidant activity for at least four years [6].

## 3. Materials and Methods

### 3.1. Plant Material

The plant material was collected from three localities of the state of Jalisco: (1) Huejuquilla (OH), located at a latitude of 22°45′ N, a length of 103°45′ O and at 1450 m above sea level; (2) Mezquitic (OM), located at 21°99′ N latitude, 103°35′ west longitude and 1380 m above sea level; and (3) Colotlan (OC), a municipality located at a latitude of 22°12′ N and a length of 103°18′ O, with an altitude of 1550 m above sea level. The botanical identification and stage of maturity of the plant material was carried out in the herbarium of the Technological Institute of Tlajomulco from the structural analysis of leaves, stems, and flowers. Plant material was classified as *Lippia graveolens* HBK with synonymy of *Lippia berlandieri* Shauer. The aerial parts of the plant were used for the study and the proportion of leaf and flowers was determined.

### 3.2. Standards

The standards of galangin (99.3%), quercetin (99.6%) and naringenin (99.4%) were purchased from Sigma-Aldrich (Darmstadt, Germany); while, pinocembrin (99.8%), eriodictyol (100%), apigenin 7-*O*-glucoside (99.1%), hispidulin (98.9%), genkwanin (99.3%), taxifolin (99.1%) and phlorizin (99%) were acquired from PhytoLab (Vestenbergsgreuth, Germany).

### 3.3. Phytochemical Analysis

The phytochemical evaluation was carried out to identify the families of compounds present in Mexican oregano samples through standard qualitative tests reported by Martínez et al. [45]. The analyzes were performed with five different extracts: water, ethanol, chloroform, 50% ethanol and 50% acetone in water from samples of crushed oregano in a ratio of 1:20 plant material: solvent, w/v. For flavonoids, 2 mL of ethanolic extract was taken and mixed with 1 mL of lead acetate to remove chlorophylls. Then, 3 to 4 magnesium (Mg) filings were added to the supernatant and finally, a few drops of concentrated HCl (Shinoda reaction) were placed. This test is positive if red and orange tints are observed. For terpenes, 1 mL of chloroform extract was taken and a few drops of acetic anhydride were added, after which concentrated H_2_SO_4_ was added (Liberman reaction). The presence of terpenes causes the extract to be colored blue-green. For steroids, 4 mL of chloroform extract were taken and a few drops of concentrated H_2_SO_4_ were added, the mixture was allowed to stand for 2 min. The formation of a reddish ring at the interface indicates the presence of steroids. For tannins, after removal of the acetone, 1 mL of extract was taken and a few drops of FeCl_3_ were added. The appearance of intense blue color indicates the presence of galotannins and elegitaninos. If the coloration is intense green shows the presence of condensed tannins. If there is the presence of both and in high concentrations, the reaction cannot be clearly distinguished and the extract will become dark or black brown. For coumarins, 1 mL of aqueous extract was taken and placed in a porcelain cap. It was mixed with few drops of NH_4_OH and finally, it visualized with UV light at 365 nm. The test is positive if blue fluorescence is observed in the extract. For quinones, 1 mL of hydroethanolic extract was extracted with a volume of petroleum ether. The organic phase was recovered and extracted again with a mixture 1:7 of ethanol: water at 60°C. The hydroalcoholic solution was separated and heated to remove the ether completely. A milliliter of 30 volume hydrogen peroxide and 1 mL of 50% sulfuric acid in water was added. The solution was cooled and then extracted with benzene. The organic phase was recovered and 1 mL of 5% NaOH (with 2% NH_4_OH) was added. It was stirred slightly. The appearance of a red ring is positive evidence of the presence of quinones. For saponins 4 mL of aqueous extract was taken in a test tube and shaken vigorously for one minute. The formation of an abundant and stable foam is a presumptive test for the presence of saponins in the sample.

### 3.4. Hydroethanolic Extraction of Phenolic Compounds

The extraction of phenolic compounds was carried out according to optimal conditions described by Flores-Martínez in previous reports, for maximum antioxidant activity [43]. The aerial parts (including leaves and flowers) of dry oregano were milled (0.4 mm) and macerated in a water-ethanol solution (ethanol 58% *v*/*v*), with a 1:20 (*w*/*v*) oregano: ethanol ratio. The extraction was done at 75 °C for one hour under magnetic stirring. The extracts were then filtered and stored at 4 °C in amber glass bottles until analysis.

### 3.5. Determination of Total Phenols

The quantification of total phenols was performed by the Folin-Ciocalteu method. The extracts were diluted to 10% and the reaction was carried out by mixing 0.5 mL of Folin 0.67 N reagent and 0.5 mL of 1.9 M Na_2_CO_3_. After 1 h, the samples were read at 760 nm. Gallic acid was used as the reference standard.

### 3.6. Antioxidant Capacity by DPPH

The antioxidant capacity of the ethanolic extracts was determined in the presence of the radical 1,1-diphenyl-2-picrylhydrazine (DPPH) at 518 nm. Two milliliters of 80% methanol (blank) and 0.1% diluted extracts were taken respectively, then 2 mL of freshly prepared 2.5 mM DPPH was added. The blank reading and samples were made after 30 min. The percent inhibition was calculated according to the following equation:

$$\%\;de\;inhibiton=\;\frac{Abs\;blank-Abs\;sample}{Abs\;blank\;}$$

The results are expressed as Trolox equivalent (TxEq) corresponding to the calibration curve (0, 20, 40, 60, 80, 100 mg TxEq /mL).

### 3.7. Antioxidant Capacity by ORAC

For the analysis of the antioxidant capacity by the ORAC method, Trolox was used as the reference standard and gallic acid as a positive control. The reaction was carried out by mixing 100 μL of 120 nM fluorescein with 20 μL of PBS (blank), Trolox or extract, respectively. The microplate and the freshly prepared 2,2′-Azobis (2-methylpropionamidine) dihydrochloride radical (AAPH) were heated at 37 °C for 15 min. Then, 80 μL of the AAPH was added to the mixture and reaction readings were taken every 4 min until the fluorescence was less than 10% of the initial fluorescence (approximately 2 h). The analysis was carried out in triplicate for each sample and level of the curve.

The fluorescence values were normalized according to the blank curve (without antioxidant). The area under the fluorescence descent curve (AUC) was calculated from the normalized curves as [46,47,48]:

(1) AUC=0.5+ f1f0+f2f0+f3f0+…+fnf0 ∗ 4

The AUC_net_ corresponding to each Trolox concentration and to each sample was calculated by subtraction of the respective AUC minus that corresponding to the blank. The regression equation was calculated according to AUC_net_ and the corresponding Trolox concentration. The ORAC-FI values were expressed as Trolox equivalents using the regression equation of the standard calibration curve.

### 3.8. Identification and Quantification of Polyphenols by LC-ESI-QTOF-MS

The identification of phenolic compounds in ethanolic extracts of *Lippia graveolens* H.B.K. was performed using an LC-ESI-QTOF-MS (Agilent) system. Separation was achieved on a Kinetex C18 column (50 × 4.6 mm ID × 2.6 microns (particle size)). The mobile phase was a mixture of water (A) and 95% acetonitrile (B), both acidified with 0.1% formic acid. The gradient program was as follows: 0–3 min 95% A, 10–17 min 0% A, 20–30 min 95% A; the flow rate was 0.4 mL/min at a temperature of 40 °C. The sample injection volume was 5 µL. The mass spectra were acquired using electrospray ionization (ESI) in negative polarity at a fragmentation voltage of 200 V. The mass spectra were recorded in a range m/z 100–1000. The flow of the drying gas was 4 mL/min, at a temperature of 300 °C, with a pressure in the nebulizer of 35 PSI and a capillary voltage of 4000 V. The data acquisition and analysis were developed with the software Galaxy Workstation, Agilent Technologies version B.04.00.

The identification of compounds was performed by three methods. The first was by direct comparison of the retention time and the pseudomolecular ion (*m*/*z*) of the peaks established the standards with those obtained from the extracts. A second identification was carried out by comparing the pseudomolecular ions previously reported by other authors and those acquired with the extracts [9,12,24,25]. The third group of compounds was identified using the “Formula generator” tool of the Qualitative Analysis-WorkStation software [12,49]. For this, both the selected spectrum of the total detected signal and the chromatogram were extracted to verify the presence of a well-defined peak. The spectrum was re-selected and the formula was generated. The formulas were recorded, their isotopic pattern was obtained and compared with the experimental molecular ion. Finally, the possible natural compounds corresponding to the formulas obtained were searched in the literature.

For the quantification of polyphenols, multi-level calibration curves were performed using standards of galangin, quercetin (flavonols), naringenin, pinocembrin, eriodictyol (flavanones), apigenin 7-*O*-glucoside, hispidulin, genkwanin (flavones), taxifolin (flavanonol) and phlorizin (chalcone). Five microliters of the sample were injected for quantification.

### 3.9. Statistical Analysis

All experiments were performed three times and data were expressed as mean ± standard deviation. The data obtained were subjected to analysis of variance (ANOVA) and comparisons between treatments were performed using Tukey test, differences were considered significant at *p* < 0.05 (STATGRAPHICS Centurion XVI version 16.1.18.).

## 4. Conclusions

The three samples of Mexican oregano present differences in their composition and phytochemical content, mainly of triterpenes and quinones. However, the obtained extracts present a total phenolic content and antioxidant activity similar, which could favor the use of this species regardless of where they come from. The chromatographic assays show that the ethanolic extracts obtained have a wide variety of compounds with an untapped importance for the development of new high value-added products. The results obtained from the phytochemical analysis, total phenols and antioxidant capacity can be used as a precedent to lay the foundations for new quality parameters and contribute to a better characterization of this important resource as a spice or as a raw material for the development of new products. For this, future studies should focus on the evaluation of a possible change in the antioxidant capacity and the qualitative and quantitative composition of the present compounds year by year.

## Figures and Tables

**Table 1:** Physiochemical and phytochemical analysis of Mexican oregano samples from 3 regions: oregano from Huejuquilla (OH), from Mezquitic (OM) and from Colotlan (OC).

Family of Compounds	OH	OM	OC
Flowering (%)	11	28	7
Leaf (%)	83	65	74
Stem (%)	4	7	18
EO (mL/100g DB)	1.4	2.6	4.8
Ripening	+++	+	++
Flavonoids	+++	+	++
Terpenes	nd	++	+++
Steroids	+	++	+++
Tannins	+++	+++	+++
Coumarins	nd	nd	nd
Quinones	nd	++	+
Saponins	+	nd	++
Alkaloids	nd	nd	nd

(+++) Strongly present, (++) moderately present, (+) weakly present, (nd) not detected.

**Table 2:** Determination of total phenols and antioxidant capacity (ORAC and DPPH) of Mexican oregano extracts from different regions of Jalisco state.

Sample	Total Phenolics (mg GA/mL Ex)	DPPH (mg TxEq/mL Ex)	ORAC (mg TxEq/mL Ex)
OH	4.41 ± 0.052 ^a^	7.54 ± 0.224 ^a^	1.66 ± 0.284 ^a^
OM	4.28 ± 0.008 ^a^	7.87 ± 0.060 ^a^	1.67 ± 0.254 ^a^
OC	4.54 ± 0.030 ^a^	8.79 ± 0.000 ^a^	1.65 ± 0.236 ^a^

OH, oregano from Huejuquilla; OM, oregano from Mezquitic; OC, oregano from Colotlan; GA, galic acid; EqTx, equivalent Trolox. Different superscripts letters indicated significant differences between samples according to Tukey test (*p* < 0.05).

**Table 3:** Identification of phenolic compounds using liquid chromatography–electrospray quadrupole-time of flight mass spectrometry (LC-ESI-QTOF/MS).

Peak	Name	Formula	m/z cal [M-H]^-^	OH		OM		OC		Reference/Specie
				m/z exp [M-H]^-^	Dif (ppm)	m/z exp [M-H]^-^	Dif (ppm)	m/z exp [M-H]^-^	Dif (ppm)	
1	Methyl 4,6-*O*-di-*O*-galloyl-β-d-glucopyranoside/Methyl 6-*O*-digalloyl-β-d-glucopyranoside I/II	C_21_ H_22_ O_14_	497.0937	497.0953	3.23	497.0941	0.79	-	-	*Sanguisorba officinalis*.
2	Tuliposide F/Cachinoside IV	C_16_ H_24_ O_11_	391.1246	391.1254	2.08	391.1243	0.51	-	-	*Tulipa turkestani/Campsis grandiflora*
3	5,7,8-trihydroxycoumarin-5-β-glucopyranoside	C_15_ H_16_ O_10_	355.0671	355.0667	0.98	-	-	-	-	*Polytrichum formosum*
4	1-*O*-(4-Hydroxybenzoyl)-β-d-glucose	C_13_ H_16_ O_8_	299.0772	299.0771	0.62	-	-	-	-	*Crocus sativus* (pollen) y *Luffa cylindrica*
5	(2*R*)- and (2*S*)-3′,4′,5,6-tetrahydroxyflavanone 7-*O*-β-glucopyranoside/(2*R*)- and (2*S*)-3′,4′,5,8-tetrahydroxyflavanone 7-*O*-β-glucopyranoside	C_21_ H_22_ O_12_	465.1022, 465.1033/465.1023, 465.1029	465.1054	3.81	-	-	-	-	[24]
6	Deacetyl asperulosidic acid/Teveside/Monotropein	C_16_ H_22_ O_11_	389.1089	389.1101	2.97	389.1087	0.02	389.1075	3.75	*Lasianthus acuminatissimus*, *Morinda citrifolia* (fruit), *Daphniphyllum macropodum*, *Lasianthus wallichi*, *Gardenia jasminoides*/*Thevetia neriifolia*, *Lippia citriodora*, *Lemon verbena*/*Cornus suecica*, *Morinda officinalis*, *Galium glaucum*, *Monotropa hypopitys*, *M. uniflora*, *Pyrola japonica*, *Arctostaphylos uva-ursi*
7	Apigenin 6,8-di-*C*-glucoside/Kaempferol-3-*O*-rutinoside	C_27_ H_30_ O_15_	593.1512	593.1537	4.11	-	-	-	-	[25,26]
8	(1*R*,2*R*)-5′-hydroxyjasmonic 5′-O-*β*-*d*-glucopiranoside acid/tuberonic glicoside acid	C_18_ H_28_ O_9_	387.1661	387.1668	2.00	387.1664	0.91	387.1646	3.89	*Thymus vulgaris*, *Perilla frutescens*, *Lippia citriodora.*
9	(2*R*)- and (2*S*)-3′,4′,5,6-tetrahydroxyflavanone 7-*O*-β-glucopyranoside/(2*R*)- and (2*S*)-3′,4′,5,8-tetrahydroxyflavanone 7-*O*-β-glucopyranoside	C_21_ H_22_ O_12_	465.1022, 465.1033/465.1023, 465.1029	465.1055	3.51	465.1048	2.02	465.1032	1.31	[24]
10	6-Hydroxyluteolin-7-*O*-hexoside	C_21_ H_20_ O_12_	463.0877	463.0899	3.65	463.0891	2.01	463.0873	2.05	[9,24]
11	(2*R*)- and (2*S*)-3′,4′,5,6-tetrahydroxyflavanone 7-*O*-β-glucopyranoside/(2*R*)- and (2*S*)-3′,4′,5,8-tetrahydroxyflavanone 7-*O*-β-glucopyranoside	C_21_ H_22_ O_12_	465.1022, 465.1033/465.1023, 465.1029	465.1050	2.54	465.1050	2.39	465.1023	3.31	[24]
12	Verbascoside/Isoverbascoside/Forsitoside A	C_29_ H_36_ O_15_	623.1981	623.2023	4.21	623.2008	3.50	623.2007	0.26	[24]
13	3-Hydroxyphloretin 6′-*O*-hexoside	C_21_ H_24_ O_11_	451.1246	-	-	451.1252	1.47	451.1241	1.18	[9]
14	(a)	C_30_ H_28_ O_15_	627.1355	-	-	627.1378	0.62	-	-	-
15	Taxifolin	C_15_ H_12_ O_7_	303.0505	303.0511	0.24	303.0503	2.36	303.0571	6.65	Standard
16	Cosmoside	C_21_ H_20_ O_10_	431.0984	431.0989	0.53	431.0989	2.37	431.0957	6.22	Standard
17	Phlorizin	C_21_ H_24_ O_10_	435.1297	435.1302	0.90	435.1307	2.27	435.1283	3.22	Standard
18	4-methoxyphenyl 1-*O*-β-d-[5-*O*-(3,4-dimethoxybenzoyl)]-apio-furanosyl-(1-6)-β-d-glucopiranoside	C_27_ H_34_ O_14_	581.1876	581.1886	1.83	-	-	-	-	*Tabebuia impetiginosa*
19	(b)	C_21_ H_24_ O_9_	419.1348	419.1347	0.13	-	-	-	-	-
20	Eriodictyol	C_15_ H_12_ O_6_	287.0569	287.0554	2.55	287.0576	6.69	287.0581	11.89	Standard
21	2′′-*O*-(3′′′,4′′′-dimethoxybenzoyl) orientin	C_30_ H_28_ O_14_	611.1406	611.1413	1.12	611.1424	2.82	611.1393	2.24	*Trollius ledebourii* (flowers).
22	Ikarisoside F	C_31_ H_36_ O_14_	631.2032	-	-	631.2057	3.85	-	-	*Epimedium koreanum*, *E. Sagittatum*, *E. pubescens*, *E. wushanense*, *E. brevicornum*
23	6′′-*O*-p-hidroxybenzoyliridyn	C_31_ H_30_ O_15_	641.1512	-	-	641.1531	2.90	-	-	*Belamcanda chinensis*
24	(c)	C_23_ H_32_ O_18_	595.1516	-	-	595.1494	3.65	595.1426	5.23	-
25	Quercetin	C_15_ H_10_ O_7_	301.0354	301.0346	1.62	301.0353	0.19	301.0329	7.59	Standard
26	Sinapic *C*-hexoside acid	C_17_ H_22_ O_10_	385.1140	385.1138	0.58	-	-	-	-	[25]
27	(d)	C_18_ H_26_ O_7_	353.1606	-	-	-	-	353.1595	3.18	-
28	Phloretin	C_15_ H_14_ O_5_	273.0763	273.0755	4.82	273.0768	0.10	273.0774	10.90	[24]
29	Naringenin	C_15_ H_12_ O_5_	271.0612	271.0604	3.05	271.0609	1.24	271.0593	6.96	Standard
30	Hispidulin	C_16_ H_12_ O_6_	299.0561	299.0551	3.35	299.0559	0.77	299.0568	7.06	Standard
31	Diterpenic taxoid	C_30_ H_42_ O_12_	593.2662/593.2604	593.2632	4.73/5.04	-	-	-	-	-
32	Cirsimaritin	C_17_ H_14_ O_6_	313.0718	313.0705	4.03	313.0718	0.17	313.0723	5.12	[9,12]
33	Pinocembrin	C_15_ H_12_ O_4_	255.0663	255.0673	3.99	255.0657	1.89	255.0644	7.50	Standard
34	Galangin	C_15_ H_10_ O_5_	269.0501	269.0463	2.76	269.0451	1.66	269.0434	8.13	Standard
35	Genkwanin	C_16_ H_12_ O_5_	283.0612	283.0605	0.01	283.0608	1.30	283.0620	8.12	Standard
36	Diterpenic quinone	C_20_ H_26_ O_3_	313.1809	-	-	313.1814	1.63	-	-	-
37	(e)	C_20_ H_24_ O_4_	327.1602	-	-	327.1599	0.71	-	-	-
38	13(*R*)-Hydroxy-octadeca-(9*Z*,11*E*,15*Z*)-trien-oic acid/Higrosforone F	C_18_ H_30_ O_3_	293.2122	293.2112	3.55	-	-	-	-	*Potamogeton lucens*/*Hygrophorus persoonii*
39	(f)	C_23_ H_46_ O_16_	577.2713	577.2690	4.00	-	-	-	-	-
40	(*S*)-cariolic acid/coronaric acid	C_18_ H_32_ O_3_	295.2279	295.2270	2.85	-	-	-	-	*Hernandia sonora*/*Chysantemun coronarium*
41	(g)	C_37_ H_28_ O_8_	599.1711	599.1706	0.97	-	-	-	-	-
42	Diterpenic quinone	C_20_ H_26_ O_3_	313.1809	-	-	-	-	313.1795	4.69	-
43	Sesquiterpene	C_15_ H_22_ O_4_	265.1445	-	-	-	-	265.1459	5.01	-
44	Chalcone	C_25_ H_28_ O_5_	407.1864	407.1858	1.41	-	-	-	-	-
45	Lancilactone B	C_30_ H_38_ O_4_	461.2691	-	-	461.2694	0.74	461.2692	1.26	*Kadsura lancilimba*
46	Diterpenic quinone	C_20_ H_26_ O_3_	313.1809	-	-	313.1808	0.53	313.1796	4.27	-
47	Lancilactone B	C_30_ H_38_ O_4_	461.2697	-	-	461.2705	1.74	-	-	*Kadsura lancilimba*
48	Diterpene	C_20_ H_24_ O_3_	311.1653	-	-	-	-	311.1641	3.78	-
49	Flavonoid	C_25_ H_28_ O_4_	391.1915	391.1902	3.35	-	-	-	-	-
50	Camaric acid	C_35_ H_52_ O_6_	567.3691	-	-	-	-	567.3688	0.23	*Lantana camara* (aerial parts), *Lantana cujabensis.*
51	Flavonoid	C_25_ H_28_ O_4_	391.1915	-	-	391.1923	2.15	-	-	-
52	Salviol	C_20_ H_30_ O_2_	301.2173	-	-	301.2172	0.48	301.2177	4.06	[12]
53	Grandidone D	C_40_ H_48_ O_8_	655.3276	-	-	-	-	655.3278	0.30	*Plectranthus grandidentatus*.
54	Flavonoid	C_25_ H_26_ O_5_	405.1707	405.1693	3.52	-	-	-	-	-
55	Lespedezol B_3_	C_40_ H_36_ O_9_	659.2287	659.2277	1.49	-	-	-	-	*Lespedeza homoloba.*
56	Lantadene A/B	C_35_ H_52_ O_5_	551.3742	551.3721	3.75	-	-	-	-	*Lantana camara*, *Cardia multispicata.*
57	Lancilactone B	C_30_ H_38_ O_4_	461.2684	-	-	-	-	461.2684	2.91	*Kadsura lancilimba*
58	Anthron	C_30_ H_36_ O_4_	459.2541	459.2519	4.53	-	-	-	-	-
59	(h)	C_19_ H_30_ O_6_	353.1969	353.1988	4.78	-	-	-	-	-
60	(i)	C_36_ H_60_ O_8_	619.4215	619.4208	0.38	-	-	-	-	-
61	(j)	C_48_ H_82_ O_5_	737.6089	-	-	737.6073	2.32	-	-	-
62	(k)	C_28_ H_44_ O_11_	555.2811	555.2845	6.22	555.2847	6.62	555.2843	4.01	-

OH: oregano from Huejuquilla, OM: oregano from Mezquitic, OC: oregano from Colotlan.

**Table 4:** Uses and properties of main phenolic compounds found in Mexican oregano extracts from different regions of Jalisco state.

Compound	Importance
Flavanones	
Pinocembrin	Naturally found in honey and propolis. Pinocembrin has shown anti-inflammatory, antioxidant, antiapoptotic, antimicrobial and vasodilator activity and antiproliferative properties [28]. There are reports of protective activity against cerebral ischemia. Likewise, there are several studies of application technologies and pharmaceutical use [29,30,31].
Naringenin	Antioxidant, anti-inflammatory, carbohydrate metabolism promoter and immune system modulator. Naringenin has high capacity to reduce plasma cholesterol level and useful for the treatment of hepatitis C [32]. Antimetastatic, naringenin stimulates DNA repair [33].
Eriodictyol	It is extracted from yerba santa (*Eriodictyon californicum*). Eriodictyol has potential use in Parkinsons treatment [9].
**Flavonols**	
Galangin	It is found in high concentrations in *Alpinia officinarum* and *Helichrysum aureonitens.* Galangin has antiviral and antibacterial properties [9].
Quercetin	It is found in high concentrations in onions. Presents antihistamine activity and antimutagenic, proapoptotic, antiangiogenic, antimetastatic properties and is a modulator of epigenetic changes [34].
**Flavones**	
Genkwanin	Antioxidant and antitumoral activities [35]. This is swine fever inhibitor [36].
Hispidulin	It has only been reported in *Rosmarinus officinalis.* Presents antihepatotoxic, cough suppressant, platelet aggregation inhibitor activities and anticancer properties in liver cells [37].
Apigenin 7-*O*-glucoside	Anti-inflammatory, antioxidant and antihemolysis [28]. Antimutagenic, pro-apoptotic, anti-angiogenic, anti-metastatic [38,39].
**Flavanonols**	
Taxifolin	Protective action of vascular system, anticancer. Promotes formation and stabilization of collagen fibrils [9].
**Chalcones**	
Phlorizin	It is found in high concentration in cultivated apples, their leaves and the bark of the roots from where it is obtained in crystalline form. It produces glycosuria in animals [9].

**Table 5:** Quantification of main phenolic compounds of Mexican oregano extracts from different regions of Jalisco state.

Compound	OH (mg/mL Ex)	OM (mg/mL Ex)	OC (mg/mL Ex)
Taxifolin	0.060 ± 0.001 ^b^	0.063 ± 0.000 ^b^	0.073 ± 0.000 ^a^
Apigenin 7-*O*-glucoside	0.015 ± 0.002 ^a^	0.009 ± 0.002 ^a^	0.008 ± 0.002 ^a^
Phlorizin	0.278 ± 0.001 ^a^	0.097 ± 0.000 ^b^	0.099 ± 0.000 ^b^
Eriodictyol	0.017 ± 0.001 ^c^	0.033 ± 0.023 ^b^	0.044 ± 0.030 ^a^
Quercetin	0.014 ± 0.001 ^a^	0.015 ± 0.007 ^a^	0.018 ± 0.008 ^a^
Naringenin	0.119 ± 0.000 ^b^	0.130 ± 0.001 ^a^	0.115 ± 0.001 ^b^
Hispidulin	0.002 ± 0.000 ^b^	0.022 ± 0.003 ^a^	0.023 ± 0.004 ^a^
Pinocembrin	3.231 + 0.390 ^a^	0.356 + 0.002 ^b^	0.020 + 0.002 ^b^
Genkwanin	0.090 ± 0.001 ^a^	0.001 + 0.000 ^b^	0.104 + 0.005 ^a^
Galangin	0.436 ± 0.020 ^c^	0.082 + 0.004 ^b^	0.003 + 0.000 ^a^

OH: oregano from Huejuquilla, OM: oregano from Mezquitic, OC: oregano from Colotlan. Different superscripts letters indicated significant differences between samples according to Tukey test (*p* < 0.05).

## Data Availability

The data presented in this study are available in this article.

## Abbreviations

AAPH	2,2′-Azobis (2-methylpropionamidine) dihydrochloride radical
AUC	area under the curve
DPPH	1,1-diphenyl-2-picrylhydrazine
GA	Galic acid
OC	Oregano from Colotlan
OH	Oregano from Huejuquilla
OM	Oregano from Mezquitic
ORAC	Oxygen Radical Absorbance Capacity
